# Vibration and current dataset of three-phase permanent magnet synchronous motors with stator faults

**DOI:** 10.1016/j.dib.2023.108952

**Published:** 2023-02-06

**Authors:** Wonho Jung, Sung-Hyun Yun, Yoon-Seop Lim, Sungjin Cheong, Yong-Hwa Park

**Affiliations:** Department of Mechanical Engineering, Korea Advanced Institute of Science and Technology, 291, Daehak-ro, Yuseong-gu, Daejeon, 34141, South Korea

**Keywords:** Three-Phase Permanent Magnet Synchronous Motors, Vibration, Current, Stator Fault, Fault Diagnosis, Condition Monitoring

## Abstract

Permanent magnet synchronous motors (PMSM) are widely used in industry applications such as home appliances, manufacturing process, high-speed trains, and electric vehicles. Unexpected faults of PMSM are directly related to the significant losses in the engineered systems. The majority of motor faults are bearing fault (mechanical) and stator fault (electrical). This article reports vibration and driving current dataset of three-phase PMSM with three different motor powers under eight different severities of stator fault. PMSM conditions including normal, inter-coil short circuit fault, and inter-turn short circuit fault in three motors are demonstrated with different powers of 1.0 kW, 1.5 kW and 3.0 kW, respectively. The PMSMs are operated under the same torque load condition and rotating speed. Dataset is acquired using one integrated electronics piezo-electric (IEPE) based accelerometer and three current transformers (CT) with National Instruments (NI) data acquisition (DAQ) board under international organization for standardization standard (ISO 10816-1:1995). Established dataset can be used to verify newly developed state-of-the-art methods for PMSM stator fault diagnosis. Mendeley Data. DOI: 10.17632/rgn5brrgrn.5

Specifications Table

**Table:** 

Subject:	Engineering – Mechanical Engineering

Specific subject area:	Fault Diagnosis of Motor Stator
Type of data:	Time-series vibration data (single-axis) Time-series driving current data (three-phases) Tables Figures
How the data were acquired:	A ceramic shear ICP based accelerometer (PCB 352C34) is mounted on motor bearing based on ISO 10816-1:1995. Three CT sensors (Hioki CT6700) are installed on U-phase, V-phase and W-phase of PMSM. Vibration data are collected from the accelerometer by NI 9234, and current data are collected from the CT sensors by NI 9775.
Data format:	Raw
Description of data collection:	The dataset was acquired from three motors with different powers of 1.0 kW, 1.5 kW and 3.0 kW, respectively. Each motor condition was experimented with PMSM with seeded artificial faults including inter-coil short circuit and inter-turn short circuit. Vibration data are collected by NI DAQ for 120 seconds at the sampling rates of 25.6 kHz. Current data are collected by NI DAQ for 120 seconds at the sampling rate of 100 kHz.
Data source location:	Institution: Human Lab., Department of Mechanical Engineering, Korea Advanced Institute of Science and Technology (KAIST) City: Daejeon Country: South Korea
Data accessibility:	Repository name: Vibration and Current Dataset of Three-Phase Permanent Magnet Synchronous Motors with Stator Faults Data identification number: 10.17632/rgn5brrgrn.5 Direct URL to data: https://data.mendeley.com/datasets/rgn5brrgrn/5

## Value of the Data

· This dataset is acquired from three motors with different powers of 1.0 kW, 1.5 kW, and 3.0 kW, respectively. Two different types of faults including inter-turn short circuits and inter-coil short circuits are seeded. This dataset consists of vibration data which represent the shock of the bearing due to torque unbalance by motor stator faults; and, current data which represent the changes of motor driving power.

· This dataset is collected according to ISO guideline (ISO 10816-1:1995). Stator faults are artificially seeded using bypassing resistances allocated in inter-coil circuits and inter-turn circuits. Three motors with different powers of 1.0 kW, 1.5 kW, and 3.0 kW are tested under the same experimental setup including rated rotating speed (3000 RPM), rated load condition (15 % of allowable torque limit of 1.5 Nm), sensor location, and their sampling rates (25.6 kHz for vibration, 100 kHz for current measurements).

· Considering dataset for fault diagnosis often requires large amounts of effort and time consumption, this dataset can provide a useful dataset in the fault diagnosis research field [1]. Therefore, this dataset can be used to analyze the condition of motor under various motor powers with different severity of motor stator faults [2,3]. Also, vibration data synchronized with the driving current data can be utilized for the analysis of the vibration-current correlation in fault diagnosis. This dataset can be used to evaluate the newly developed deep learning based fault diagnosis method that deals with different motor powers [4].

## 1 Objective

This dataset was established for deep learning based motor fault diagnosis research. Unlike other studies, it is very difficult to obtain data in the fault diagnosis research field because it is difficult to apply an actual failure. Therefore, there are many difficulties in training of deep learning algorithm. To solve this problem, we simulated motor stator faults according to the motor power, and obtained vibration and driving current data according to the severity of their faults. This dataset is measured based on mechanical engineering knowledge in accordance with ISO international standards. This dataset used for verification of the deep learning based fault diagnosis method.

## 2 Data description

Collected dataset consists of vibration and current data acquired from the three PMSMs with different powers of 1.0 kW, 1.5 kW and 3.0 kW. In each motor, total 16 stator faults are seeded with 8 inter-coil circuit faults and 8 inter-turn circuit faults. The motors rotate at a rated rotating speed of 3000 RPM and rated load condition (15 % of allowable torque limit of 1.5 Nm). The collected dataset is stored in technical data management streaming (TDMS) files. TDMS file format can be accessed easily with other data analysis program such as MATLAB [5], and Python [6].

Vibration data were measured using accelerometer (PCB352C34) and acquired using NI9234 module for 120 seconds with sampling frequency of 25.6 kHz. Each vibration data file contains two columns namely ‘Time Stamp’, and ‘amplitude’. The unit of the vibration amplitude is ‘gravitational constant (g)’ (1g = 9.80665 m/s^2^). The vibration data file includes z-direction of PMSM for inter-turn short circuit and inter-coil short circuit. The description of the vibration files as per operating and health conditions of the motor are provided as follows:

1. 1000W_0_00_vibration_interturn.tdms: This file includes healthy inter-turn short circuit vibration data in z-direction acquired from the motor whose power is 1.0 kW.
2. 1000W_2_26_vibration_interturn.tdms: This file includes inter-turn short circuit fault vibration data in z-direction with 2.26 % severity acquired from the motor whose power is 1.0 kW.
3. 1000W_2_70_vibration_interturn.tdms: This file includes inter-turn short circuit fault vibration data in z-direction with 2.70 % severity acquired from the motor whose power is 1.0 kW.
4. 1000W_3_35_vibration_interturn.tdms: This file includes inter-turn short circuit fault vibration data in z-direction with 3.35 % severity acquired from the motor whose power is 1.0 kW.
5. 1000W_4_41_vibration_interturn.tdms: This file includes inter-turn short circuit fault vibration data in z-direction with 4.41 % severity acquired from the motor whose power is 1.0 kW.
6. 1000W_6_48_vibration_interturn.tdms: This file includes inter-turn short circuit fault vibration data in z-direction with 6.48 % severity acquired from the motor whose power is 1.0 kW.
7. 1000W_12_17_vibration_interturn.tdms: This file includes inter-turn short circuit fault vibration data in z-direction with 12.17 % severity acquired from the motor whose power is 1.0 kW.
8. 1000W_21_69_vibration_interturn.tdms: This file includes inter-turn short circuit fault vibration data in z-direction with 21.69 % severity acquired from the motor whose power is 1.0 kW.
9. 1000W_0_00_vibration_intercoil.tdms: This file includes healthy inter-coil short circuit vibration data in z-direction acquired from the motor whose power is 1.0 kW.
10. 1000W_0_68_vibration_intercoil.tdms: This file includes inter-coil short circuit fault vibration data in z-direction with 0.68 % severity acquired from the motor whose power is 1.0 kW.
11. 1000W_0_81_vibration_intercoil.tdms: This file includes inter-coil short circuit fault vibration data in z-direction with 0.81 % severity acquired from the motor whose power is 1.0 kW.
12. 1000W_1_01_vibration_intercoil.tdms: This file includes inter-coil short circuit fault vibration data in z-direction with 1.01 % severity acquired from the motor whose power is 1.0 kW.
13 1000W_1_34_vibration_intercoil.tdms: This file includes inter-coil short circuit fault vibration data in z-direction with 1.34 % severity acquired from the motor whose power is 1.0 kW.
14. 1000W_2_00_vibration_intercoil.tdms: This file includes inter-coil short circuit fault vibration data in z-direction with 2.00 % severity acquired from the motor whose power is 1.0 kW.
15. 1000W_3_93_vibration_intercoil.tdms: This file includes inter-coil short circuit fault vibration data in z-direction with 3.93 % severity acquired from the motor whose power is 1.0 kW.
16. 1000W_7_56_vibration_intercoil.tdms: This file includes inter-coil short circuit fault vibration data in z-direction with 7.56 % severity acquired from the motor whose power is 1.0 kW.
17. 1500W_0_00_vibration_interturn.tdms: This file includes healthy inter-turn short circuit vibration data in z-direction acquired from the motor whose power is 1.5 kW.
18. 1500W_1_57_vibration_interturn.tdms: This file includes inter-turn short circuit fault vibration data in z-direction with 1.57 % severity acquired from the motor whose power is 1.5 kW.
19. 1500W_1_88_vibration_interturn.tdms: This file includes inter-turn short circuit fault vibration data in z-direction with 1.88 % severity acquired from the motor whose power is 1.5 kW.
20. 1500W_2_34_vibration_interturn.tdms: This file includes inter-turn short circuit fault vibration data in z-direction with 2.34 % severity acquired from the motor whose power is 1.5 kW.
21. 1500W_3_10_vibration_interturn.tdms: This file includes inter-turn short circuit fault vibration data in z-direction with 3.10 % severity acquired from the motor whose power is 1.5 kW.
22. 1500W_4_57_vibration_interturn.tdms: This file includes inter-turn short circuit fault vibration data in z-direction with 4.57 % severity acquired from the motor whose power is 1.5 kW.
23. 1500W_8_74_vibration_interturn.tdms: This file includes inter-turn short circuit fault vibration data in z-direction with 8.74 % severity acquired from the motor whose power is 1.5 kW.
24. 1500W_16_08_vibration_interturn.tdms: This file includes inter-turn short circuit fault vibration data in z-direction with 16.08 % severity acquired from the motor whose power is 1.5 kW.
25. 1500W_0_00_vibration_intercoil.tdms: This file includes healthy inter-coil short circuit vibration data in z-direction acquired from the motor whose power is 1.5 kW.
26. 1500W_4_79_vibration_intercoil.tdms: This file includes inter-coil short circuit fault vibration data in z-direction with 4.79 % severity acquired from the motor whose power is 1.5 kW.
27. 1500W_5_70_vibration_intercoil.tdms: This file includes inter-coil short circuit fault vibration data in z-direction with 5.70 % severity acquired from the motor whose power is 1.5 kW.
28. 1500W_7_02_vibration_intercoil.tdms: This file includes inter-coil short circuit fault vibration data in z-direction with 7.02 % severity acquired from the motor whose power is 1.5 kW.
29. 1500W_9_15_vibration_intercoil.tdms: This file includes inter-coil short circuit fault vibration data in z-direction with 9.15 % severity acquired from the motor whose power is 1.5 kW.
30. 1500W_13_12_vibration_intercoil.tdms: This file includes inter-coil short circuit fault vibration data in z-direction with 13.12 % severity acquired from the motor whose power is 1.5 kW.
31. 1500W_23_20_vibration_intercoil.tdms: This file includes inter-coil short circuit fault vibration data in z-direction with 23.20 % severity acquired from the motor whose power is 1.5 kW.
32. 1500W_37_66_vibration_intercoil.tdms: This file includes inter-coil short circuit fault vibration data in z-direction with 37.66 % severity acquired from the motor whose power is 1.5 kW.
33. 3000W_0_00_vibration_interturn.tdms: This file includes healthy inter-turn short circuit vibration data in z-direction acquired from the motor whose power is 3.0 kW.
34. 3000W_1_78_vibration_interturn.tdms: This file includes inter-turn short circuit fault vibration data in z-direction with 1.78 % severity acquired from the motor whose power is 3.0 kW.
35. 3000W_2_13_vibration_interturn.tdms: This file includes inter-turn short circuit fault vibration data in z-direction with 2.13 % severity acquired from the motor whose power is 3.0 kW.
36. 3000W_2_65_vibration_interturn.tdms: This file includes inter-turn short circuit fault vibration data in z-direction with 2.65 % severity acquired from the motor whose power is 3.0 kW.
37. 3000W_3_50_vibration_interturn.tdms: This file includes inter-turn short circuit fault vibration data in z-direction with 3.50 % severity acquired from the motor whose power is 3.0 kW.
38. 3000W_5_16_vibration_interturn.tdms: This file includes inter-turn short circuit fault vibration data in z-direction with 5.16 % severity acquired from the motor whose power is 3.0 kW.
39. 3000W_9_81_vibration_interturn.tdms: This file includes inter-turn short circuit fault vibration data in z-direction with 9.81 % severity acquired from the motor whose power is 3.0 kW.
40. 3000W_17_86_vibration_interturn.tdms: This file includes inter-turn short circuit fault vibration data in z-direction with 17.86 % severity acquired from the motor whose power is 3.0 kW.
41. 3000W_0_00_vibration_intercoil.tdms: This file includes healthy inter-coil short circuit vibration data in z-direction acquired from the motor whose power is 3.0 kW.
42. 3000W_2_49_vibration_intercoil.tdms: This file includes inter-coil short circuit fault vibration data in z-direction with 2.49 % severity acquired from the motor whose power is 3.0 kW.
43. 3000W_2_98_vibration_intercoil.tdms: This file includes inter-coil short circuit fault vibration data in z-direction with 2.98 % severity acquired from the motor whose power is 3.0 kW.
44. 3000W_3_69_vibration_intercoil.tdms: This file includes inter-coil short circuit fault vibration data in z-direction with 3.69 % severity acquired from the motor whose power is 3.0 kW.
45. 3000W_4_86_vibration_intercoil.tdms: This file includes inter-coil short circuit fault vibration data in z-direction with 4.86 % severity acquired from the motor whose power is 3.0 kW.
46. 3000W_7_12_vibration_intercoil.tdms: This file includes inter-coil short circuit fault vibration data in z-direction with 7.12 % severity acquired from the motor whose power is 3.0 kW.
47. 3000W_13_10_vibration_intercoil.tdms: This file includes inter-coil short circuit fault vibration data in z-direction with 13.10 % severity acquired from the motor whose power is 3.0 kW.
48. 3000W_23_48_vibration_intercoil.tdms: This file includes inter-coil short circuit fault vibration data in z-direction with 23.48 % severity acquired from the motor whose power is 3.0 kW.

Current data were measured using three CT sensors named Hioki CT6700 and acquired using an NI9775 module for 120 seconds with a sampling frequency of 100 kHz. Each driving current data file contains four columns namely ‘Time Stamp’, ‘U-phase’, ‘V-phase’, and ‘W-phase’. The unit of the current is ‘ampere (A)’. The description of the current files as per operating and health conditions of the motor are provided as follows:

1. 1000W_0_00_current_interturn.tdms: This file includes healthy inter-turn short circuit current data in three phases acquired from the motor whose power is 1.0 kW.
2. 1000W_2_26_current_interturn.tdms: This file includes inter-turn short circuit fault current data in three phases with 2.26 % severity acquired from the motor whose power is 1.0 kW.
3. 1000W_2_70_current_interturn.tdms: This file includes inter-turn short circuit fault current data in three phases with 2.70 % severity acquired from the motor whose power is 1.0 kW.
4. 1000W_3_35_current_interturn.tdms: This file includes inter-turn short circuit fault current data in three phases with 3.35 % severity acquired from the motor whose power is 1.0 kW.
5. 1000W_4_41_current_interturn.tdms: This file includes inter-turn short circuit fault current data in three phases with 4.41 % severity acquired from the motor whose power is 1.0 kW.
6. 1000W_6_48_current_interturn.tdms: This file includes inter-turn short circuit fault current data in three phases with 6.48 % severity acquired from the motor whose power is 1.0 kW.
7. 1000W_12_17_current_interturn.tdms: This file includes inter-turn short circuit fault current data in three phases with 12.17 % severity acquired from the motor whose power is 1.0 kW.
8. 1000W_21_69_current_interturn.tdms: This file includes inter-turn short circuit fault current data in three phases with 21.69 % severity acquired from the motor whose power is 1.0 kW.
9. 1000W_0_00_current_intercoil.tdms: This file includes healthy inter-coil short circuit current data in three phases acquired from the motor whose power is 1.0 kW.
10. 1000W_0_68_current_intercoil.tdms: This file includes inter-coil short circuit fault current data in three phases with 0.68 % severity acquired from the motor whose power is 1.0 kW.
11. 1000W_0_81_current_intercoil.tdms: This file includes inter-coil short circuit fault current data in three phases with 0.81 % severity acquired from the motor whose power is 1.0 kW.
12. 1000W_1_01_current_intercoil.tdms: This file includes inter-coil short circuit fault current data in three phases with 1.01 % severity acquired from the motor whose power is 1.0 kW.
13. 1000W_1_34_current_intercoil.tdms: This file includes inter-coil short circuit fault current data in three phases with 1.34 % severity acquired from the motor whose power is 1.0 kW.
14. 1000W_2_00_current_intercoil.tdms: This file includes inter-coil short circuit fault current data in three phases with 2.00 % severity acquired from the motor whose power is 1.0 kW.
15. 1000W_3_93_current_intercoil.tdms: This file includes inter-coil short circuit fault current data in three phases with 3.93 % severity acquired from the motor whose power is 1.0 kW.
16. 1000W_7_56_current_intercoil.tdms: This file includes inter-coil short circuit fault current data in three phases with 7.56 % severity acquired from the motor whose power is 1.0 kW.
17. 1500W_0_00_current_interturn.tdms: This file includes healthy inter-turn short circuit current data in three phases acquired from the motor whose power is 1.5 kW.
18. 1500W_1_57_current_interturn.tdms: This file includes inter-turn short circuit fault current data in three phases with 1.57 % severity acquired from the motor whose power is 1.5 kW.
19. 1500W_1_88_current_interturn.tdms: This file includes inter-turn short circuit fault current data in three phases with 1.88 % severity acquired from the motor whose power is 1.5 kW.
20. 1500W_2_34_current_interturn.tdms: This file includes inter-turn short circuit fault current data in three phases with 2.34 % severity acquired from the motor whose power is 1.5 kW.
21. 1500W_3_10_current_interturn.tdms: This file includes inter-turn short circuit fault current data in three phases with 3.10 % severity acquired from the motor whose power is 1.5 kW.
22. 1500W_4_57_current_interturn.tdms: This file includes inter-turn short circuit fault current data in three phases with 4.57 % severity acquired from the motor whose power is 1.5 kW.
23. 1500W_8_74_current_interturn.tdms: This file includes inter-turn short circuit fault current data in three phases with 8.74 % severity acquired from the motor whose power is 1.5 kW.
24. 1500W_16_08_current_interturn.tdms: This file includes inter-turn short circuit fault current data in three phases with 16.08 % severity acquired from the motor whose power is 1.5 kW.
25. 1500W_0_00_current_intercoil.tdms: This file includes healthy inter-coil short circuit current data in three phases acquired from the motor whose power is 1.5 kW.
26. 1500W_4_79_current_intercoil.tdms: This file includes inter-coil short circuit fault current data in three phases with 4.79 % severity acquired from the motor whose power is 1.5 kW.
27. 1500W_5_70_current_intercoil.tdms: This file includes inter-coil short circuit fault current data in three phases with 5.70 % severity acquired from the motor whose power is 1.5 kW.
28. 1500W_7_02_current_intercoil.tdms: This file includes inter-coil short circuit fault current data in three phases with 7.02 % severity acquired from the motor whose power is 1.5 kW.
29. 1500W_9_15_current_intercoil.tdms: This file includes inter-coil short circuit fault current data in three phases with 9.15 % severity acquired from the motor whose power is 1.5 kW.
30. 1500W_13_12_current_intercoil.tdms: This file includes inter-coil short circuit fault current data in three phases with 13.12 % severity acquired from the motor whose power is 1.5 kW.
31. 1500W_23_20_current_intercoil.tdms: This file includes inter-coil short circuit fault current data in three phases with 23.20 % severity acquired from the motor whose power is 1.5 kW.
32. 1500W_37_66_current_intercoil.tdms: This file includes inter-coil short circuit fault current data in three phases with 37.66 % severity acquired from the motor whose power is 1.5 kW.
33. 3000W_0_00_current_interturn.tdms: This file includes healthy inter-turn short circuit current data in three phases acquired from the motor whose power is 3.0 kW.
34. 3000W_1_78_current_interturn.tdms: This file includes inter-turn short circuit fault current data in three phases with 1.78 % severity acquired from the motor whose power is 3.0 kW.
35. 3000W_2_13_current_interturn.tdms: This file includes inter-turn short circuit fault current data in three phases with 2.13 % severity acquired from the motor whose power is 3.0 kW.
36. 3000W_2_65_current_interturn.tdms: This file includes inter-turn short circuit fault current data in three phases with 2.65 % severity acquired from the motor whose power is 3.0 kW.
37. 3000W_3_50_current_interturn.tdms: This file includes inter-turn short circuit fault current data in three phases with 3.50 % severity acquired from the motor whose power is 3.0 kW.
38. 3000W_5_16_current_interturn.tdms: This file includes inter-turn short circuit fault current data in three phases with 5.16 % severity acquired from the motor whose power is 3.0 kW.
39. 3000W_9_81_current_interturn.tdms: This file includes inter-turn short circuit fault current data in three phases with 9.81 % severity acquired from the motor whose power is 3.0 kW.
40. 3000W_17_86_current_interturn.tdms: This file includes inter-turn short circuit fault current data in three phases with 17.86 % severity acquired from the motor whose power is 3.0 kW.
41. 3000W_0_00_current_intercoil.tdms: This file includes healthy inter-coil short circuit current data in three phases acquired from the motor whose power is 3.0 kW.
42. 3000W_2_49_current_intercoil.tdms: This file includes inter-coil short circuit fault current data in three phases with 2.49 % severity acquired from the motor whose power is 3.0 kW.
43. 3000W_2_98_current_intercoil.tdms: This file includes inter-coil short circuit fault current data in three phases with 2.98 % severity acquired from the motor whose power is 3.0 kW.
44. 3000W_3_69_current_intercoil.tdms: This file includes inter-coil short circuit fault current data in three phases with 3.69 % severity acquired from the motor whose power is 3.0 kW.
45. 3000W_4_86_current_intercoil.tdms: This file includes inter-coil short circuit fault current data in three phases with 4.86 % severity acquired from the motor whose power is 3.0 kW.
46. 3000W_7_12_current_intercoil.tdms: This file includes inter-coil short circuit fault current data in three phases with 7.12 % severity acquired from the motor whose power is 3.0 kW.
47. 3000W_13_10_current_intercoil.tdms: This file includes inter-coil short circuit fault current data of three phases with 13.10 % severity acquired from the motor whose power is 3.0 kW.
48. 3000W_23_48_current_intercoil.tdms: This file includes inter-coil short circuit fault current data in three phases with 23.48 % severity acquired from the motor whose power is 3.0 kW.

## 3 Experimental Design, Materials and Methods

### 3.1 Section 1: Description of Testbed

**Fig. 1:**
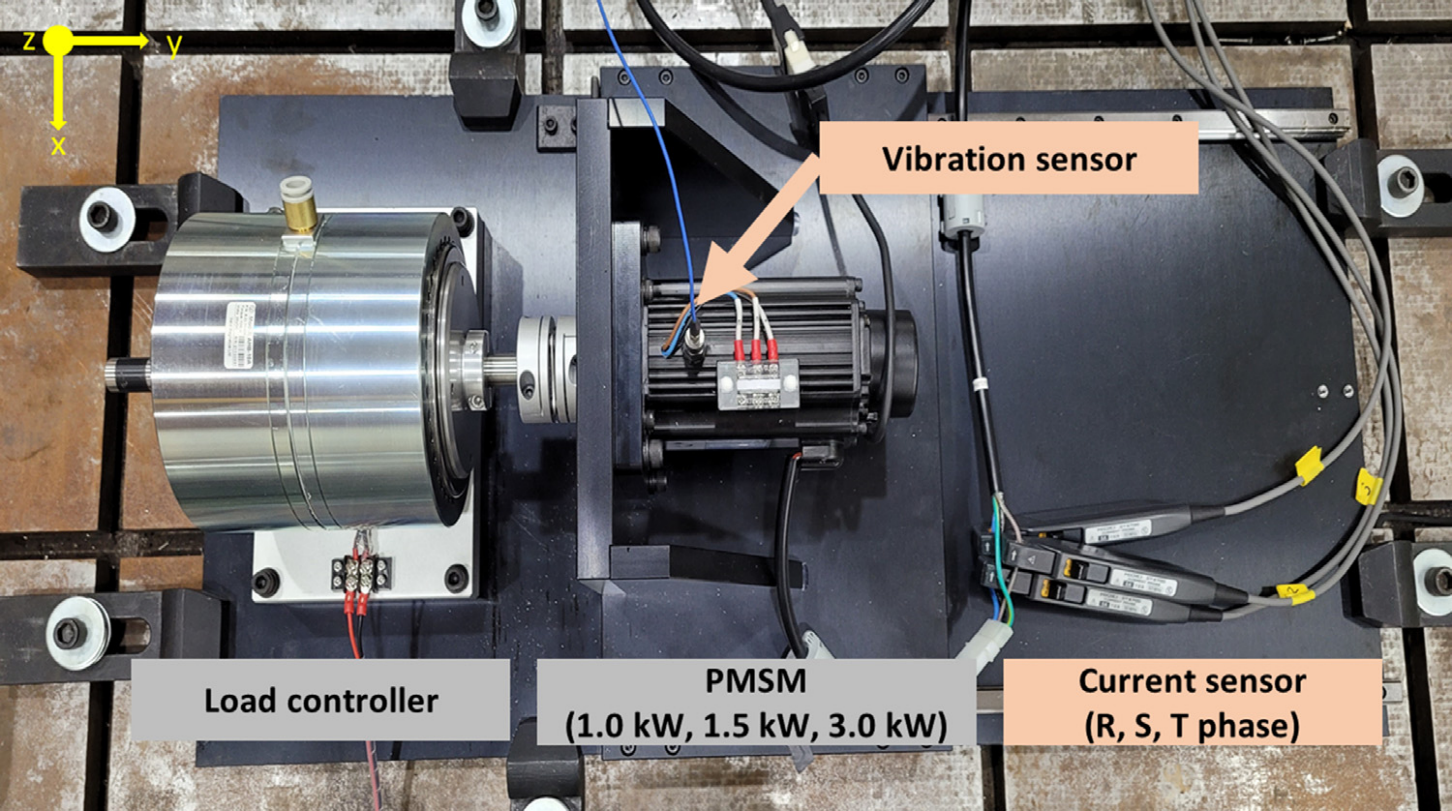
Description of PMSM testbed

PMSM testbed was built to measure the vibration and current data of healthy state and faulty states from PMSMs with different powers as shown in Fig. 1. The testbed includes a load controller made by hysteresis brake, PMSM, and sensors. Hysteresis brake (AHB-10A) manufactured by Valid Magnetics Ltd., can apply a torque load up to 10 Nm to the PMSM. Flexible couplings and a linear guide were used to prevent misalignment faults in connection between the load controller and PMSM. Parameters of PMSMs used in the experiment are summarized in Table 1. Three PMSMs are three-phase with four pole motor, and also have the same manufacturer, the same operating condition of rotating speed (3000 RPM), and 15 % of allowable torque limit of 1.5 Nm, but different powers (1.0 kW, 1.5 kW, and 3.0 kW).

**Table 1:** Parameters of PMSM

Parameters	1^st^ PMSM (1.0 kW)	2^nd^ PMSM (1.5 kW)	3^rd^ PMSM (3.0 kW)	Unit
Manufacturing company	Higen motors	Higen motors	Higen motors	-
Rated power	1000	1500	3000	Watt
Input voltage	380	380	380	AC Voltage
Frequency	60	60	60	Hz
Number of phase	3	3	3	Phase
Number of pole	4	4	4	-
Rated torque	3.18	4.77	9.55	Nm
Rated speed	3000	3000	3000	RPM
Synchronous inductance	0.	0.	0.	H
Magnetic flux	400	350	300	mT
Rotor inertia	2.07	7.48	14.34	Kgm^2^
Inter-turn resistance value (*R_it_*)	0.1385	0.0958	0.1087	Ohm
Inter-coil resistance value (*R_cc_*)	0.0409	0.3021	0.1534	Ohm

### 3.2 Section 2: Method of Fault Seedings

Failure modes of the motor stator can be categorized into: first, the increase of the resistance of the stator (open-like fault); or second, the decrease of the resistance of the stator (short-like fault). In case of open-like fault, a part of the state coil is damaged increasing the stator resistance so that driving current decreased proportional to the resistance change for given input voltage. Hence this type of failure is relatively easy to detect by reading the overall decrease of the current. On the other hand, most of difficult failures come from the second failure mode such that part of stator coil is damaged to have a short-like circuit between turns (so called inter-turn short circuit fault); or between coils (so called inter-coil short circuit fault in this work). These type of short circuit faults introduce a bypassing path of the driving current so that the normal current flowing through the stator coil is reduced by the Kirchhoff's law. As result, the motor experiences reduction of electro-magnetic field in turn the induced torque as well. Considering these types of short circuit faults, we seeded inter-turn short circuit fault and inter-coil short circuit fault controlling the bypass resistances in the short circuits as follows.

**Fig. 2:**
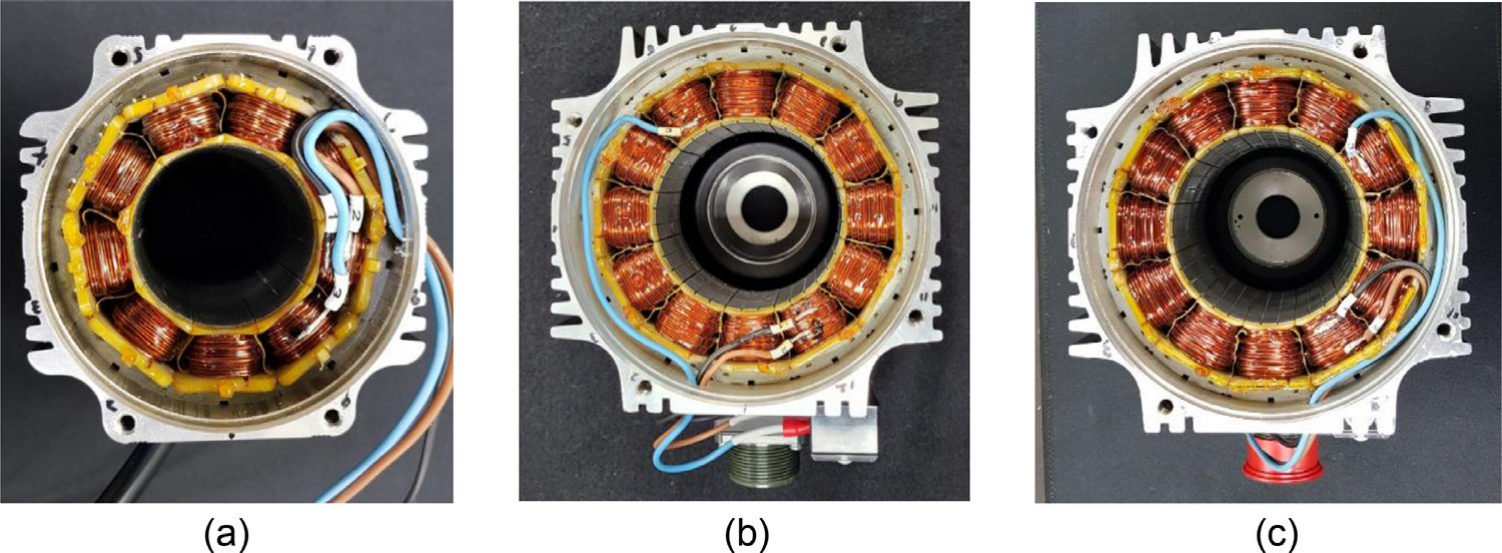
Artificial fault seeded on U-phase of PMSMs with different powers: (a) 1.0 kW, (b) 1.5 kW, and (c) 3.0 kW

**Fig. 3:**
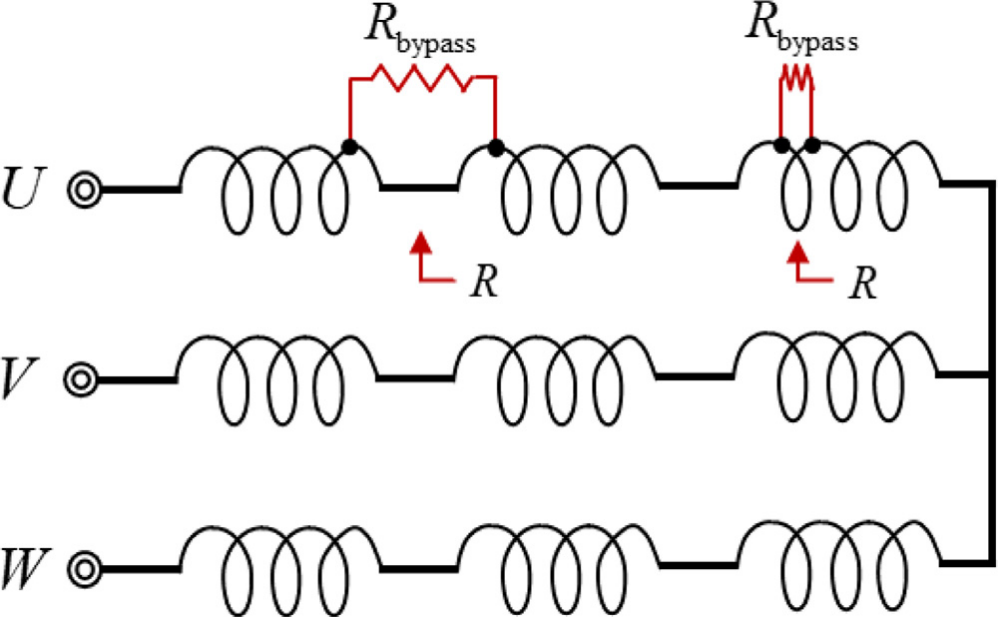
Stator winding of short circuit fault scheme where *R*_bypass_ is bypassing resistance, and *R* is stator circuit resistance

The smaller the bypass resistance value, the larger driving current flows to the bypass resistance, then the smaller driving current flows through the motor stator due to Kirchhoff's law. In this case, the severity of the motor's fault is regarded as high. Therefore, the fault was representatively seeded by adding a short circuit connecting turns (or coils) using the bypassing resistance to the motor winding as shown in Fig 2. Inter-turn short circuit faults were artificially seeded between first and second turn of U-phase of PMSM, and inter-coil short circuit is also artificially seeded between the last and the first turns of consecutive U-phases to acquired faulty data as shown in Fig. 3. The severities of inter-turn short circuit fault and inter-coil short circuit fault are calculated by Eq. (1). As the amount of the current in the bypassing circuit increases, the amount of driving current through the motor stator is accordingly reduced, therefore, the fault ratio (FR) is defined by the ratio of the amount of current in the bypassing circuit to the normal circuit. The overall description of dataset is summarized in Table 2.

(1) $$\text{Fault}\text{Ratio}(\text{FR})=\left(\frac{R}{R+R_{\text{bypass}}}\right)\times 100$$

where *R*_bypass_ is the bypassing resistance values, and the *R* is stator resistance values.

**Table 2:** Description of dataset

Data types	Sampling rate (kHz)	Length (second)	Rotating speed (RPM)	Fault types	bypassing resistance (ohm)	Fault ratio (%)		
						1^st^ PMSM (1.0 kW)	2^nd^ PMSM (1.5 kW)	3^rd^ PMSM (3.0 kW)
Vibration, current	25.6, 100	120	3000	Inter-turn short circuit	0	0 (normal)	0 (normal)	0 (normal)
					
					6	2.26	1.57	1.78
					
					5	2.70	1.88	2.13
					
					4	3.35	2.34	2.65
					
					3	4.41	3.10	3.50
					
					2	6.48	4.57	5.16
					
					1	12.17	8.74	9.81
					
					0.5	21.69	16.08	17.86
				
				Inter-coil short circuit	0	0 (normal)	0 (normal)	0 (normal)
					
					6	0.68	4.79	2.49
					
					5	0.81	5.70	2.98
					
					4	1.01	7.02	3.69
					
					3	1.34	9.15	4.86
					2	2.00	13.12	7.12
					
					1	3.93	23.20	13.30
					
					0.5	7.56	37.66	23.48

## Ethics Statements

Human Lab., Department of Mechanical Engineering, Korea Advanced Institute of Science and Technology, Daejeon, South Korea has given the consent that the datasets may be publicly-released as part of this publication. We declare that the manuscript adheres to Ethics in publishing standards and the submitted dataset is the real data recorded in the experiment, and there is no act of stealing other people's data or modifying data.

## CRediT authorship contribution statement

**Wonho Jung:** Conceptualization, Methodology, Software, Validation, Visualization, Data curation, Writing – original draft, Writing – review & editing. **Sung-Hyun Yun:** Data curation. **Yoon-Seop Lim:** Investigation. **Sungjin Cheong:** Investigation. **Yong-Hwa Park:** Funding acquisition, Writing – review & editing, Supervision.

## Declaration of Competing Interest

The authors declare that they have no known competing financial interests or personal relationships that could have appeared to influence the work reported in this paper.

## Data Availability

Vibration and Current Dataset of Three-Phase Permanent Magnet Synchronous Motors with Stator Faults (Original data) (Mendeley Data).

## References

[1] Kumar D., Mehran S., Shaikh M.Z., Hussain M., Chowdhry B.S., Hussain T. (2022). Triaxial bearing vibration dataset of induction motor under varying load condition. Data Brief.

[2] Gangsar P., Tiwari R. (2020). Signal based condition monitoring techniques for fault detection and diagnosis of induction motors: a state-of-the-art review. Mech. Syst. Signal Process..

[3] Shifat T.A., Hur J.W. (2020). An Effective Stator Fault Diagnosis Framework of BLDC Motor Based on Vibration and Current Signals. IEEE Access.

[4] Yang Y., Haque M.M., Bai D., Tang W. (2021). Fault Diagnosis of Electric Motors Using Deep Learning Algorithms and Its Application: A Review. Energy.

[5] MATLAB documentation of TDMS Format Files, Matworks official site. https://kr.mathworks.com/help/daq/tdms-format-files.html, 2022 (accessed 22.08.11)

[6] npTDMS's documentation for Python, npTDMS official site. https://nptdms.readthedocs.io/en/stable/index.html, 2022 (accessed 22.08.11)

